# Cuproptosis: mechanisms and links with cancers

**DOI:** 10.1186/s12943-023-01732-y

**Published:** 2023-03-07

**Authors:** Jiaming Xie, Yannan Yang, Yibo Gao, Jie He

**Affiliations:** 1 Department of Thoracic Surgery, National Cancer Center/National Clinical Research Center for Cancer/Cancer Hospital, Chinese Academy of Medical Sciences and Peking Union Medical College, Beijing, 100021 China; 2 State Key Laboratory of Molecular Oncology, National Cancer Center, National Clinical Research Center for Cancer, Cancer Hospital, Chinese Academy of Medical Sciences and Peking Union Medical College, Beijing, 100021 China; 3 Central Laboratory & Shenzhen Key Laboratory of Epigenetics and Precision Medicine for Cancers, National Cancer Center/National Clinical Research Center for Cancer/Cancer Hospital & Shenzhen Hospital, Chinese Academy of Medical Sciences and Peking Union Medical College, Shenzhen, 518116 China; 4 Laboratory of Translational Medicine, National Cancer Center/National, Clinical Research Center for Cancer/Cancer Hospital, Chinese Academy of Medical Sciences and Peking Union Medical College, Beijing, 101399 China

**Keywords:** Cuproptosis, Copper, Cancer, Targeted therapy, Immunotherapy, Drug resistance, Metabolism

## Abstract

Cuproptosis was a copper-dependent and unique kind of cell death that was separate from existing other forms of cell death. The last decade has witnessed a considerable increase in investigations of programmed cell death, and whether copper induced cell death was an independent form of cell death has long been argued until mechanism of cuproptosis has been revealed. After that, increasing number of researchers attempted to identify the relationship between cuproptosis and the process of cancer. Thus, in this review, we systematically detailed the systemic and cellular metabolic processes of copper and the copper-related tumor signaling pathways. Moreover, we not only focus on the discovery process of cuproptosis and its mechanism, but also outline the association between cuproptosis and cancers. Finally, we further highlight the possible therapeutic direction of employing copper ion ionophores with cuproptosis-inducing functions in combination with small molecule drugs for targeted therapy to treat specific cancers.

## Background

**Fig. 1:**
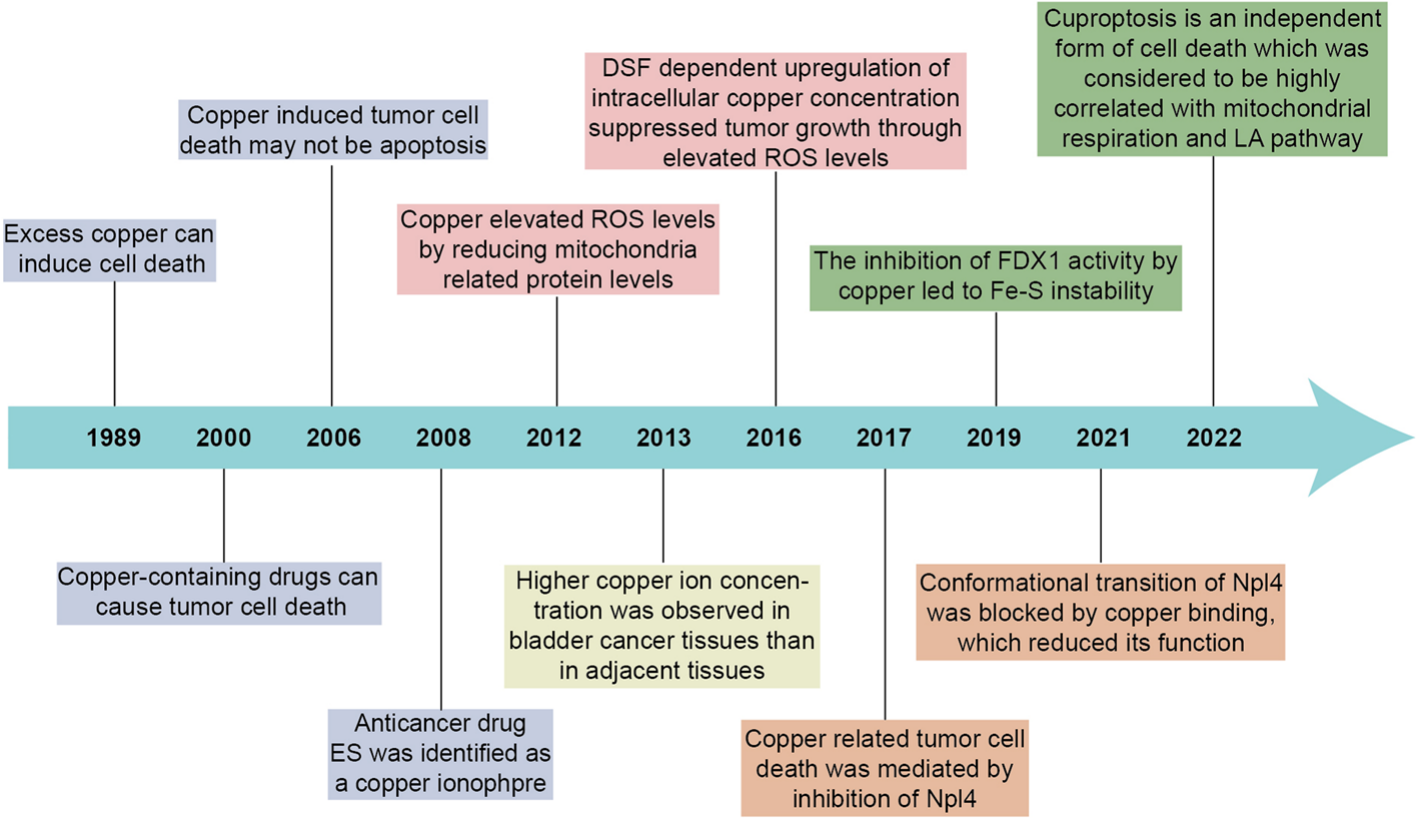
Timeline illustrating the discovery of cuproptosis. The historical events contributing to the discovery of cuproptopsis and oncological research advances of copper associated cell death are depicted in the timeline

In the recent years, cuproptosis, a novel form of regulated cell death which is copper dependent has been identified [1, 2], may be implicated in the process of various cancers. Copper is a trace element in the human body and has been strongly associated with various signaling pathways and tumor-related biological behaviors [3]. Moreover, excess copper can lead to cell death, and for a long time the mechanisms and specific forms of copper-induced cell death have remained unclear. Until early this year, it has been suggested by a recent study that cuproptosis is an independent form of cell death, which was considered to be highly correlated with mitochondrial respiration and lipoic acid(LA) pathway [4]. We briefly summarize some of the findings on copper-induced cell death that have driven progress in the field (Fig. 1).

A considerable number of researchers focusing on the pivotal relationship between cuproptosis and cancers. On the one hand, cancer has multiple types, with sufficient multi-omics data. On the other hand, cuproptosis is highly related to cellular metabolism, and certain cancer types usually exhibits high aerobic respiration levels. Some tumor types such as melanoma, breast cancer and leukemia [5, 6], some cancers with tumor stem cells [7, 8] and some drug-resistant tumors exhibit a high mitochondrial metabolic state [9–13]. Tumor cells treated with certain antitumor drugs such as proteasome inhibitors(PI) have also been found to exhibit higher mitochondrial metabolism [14, 15]. A growing number of researchers focusing on the vital link between cuproptosis and cancer process through bioinformatic analysis. Some studies have focused on the relationship between expression levels of cuproptosis key genes (CKGs), genes identified and validated in the previous studies of Tsvetkov et al., and tumor prognosis. To avoid the effects of gene interactions, some investigators have constructed Cuproptosis-related signatures by cuproptosis related genes (CRGs) to identify the association of Cuproptosis with cancer. Copper ionophores played a major contribution in the discovery of cuproptosis, and have been considered for possible use in antitumor therapy in the past [16, 17]. However, their specific mechanisms and applicable populations have not been fully analyzed. With the discovery of the cuproptosis, the interactions between these drugs, copper and the mitochondria are becoming clear, which makes the antitumor clinical application of these drugs possible. This review focusing on discovery of the mechanism of cuproptosis and the pivotal relationship between cuproptosis and cancers. We aimed to provide possible directions for future studies related to cuproptosis and cancers.

### Systemic and cellular copper homeostasis

**Fig. 2:**
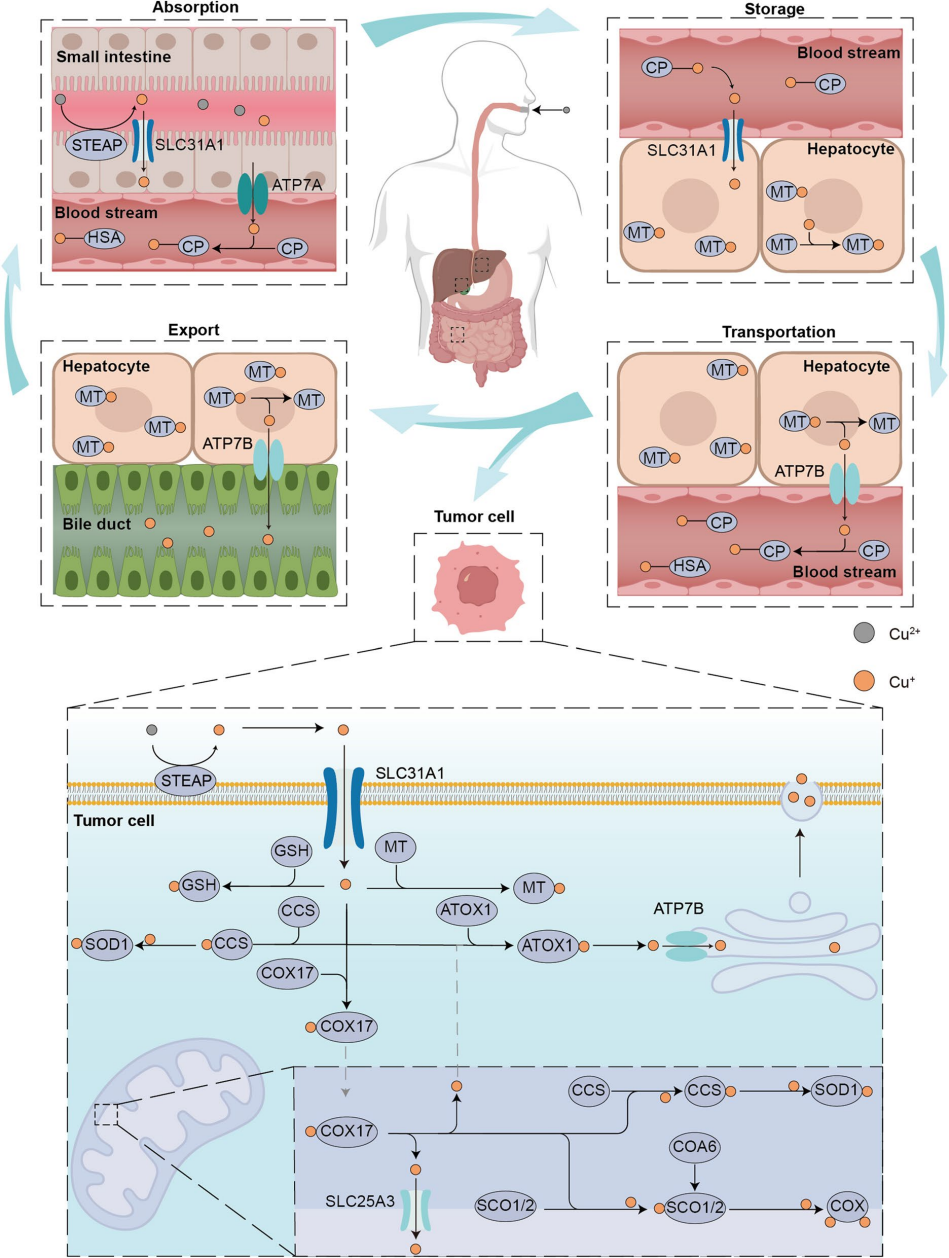
Schematic of systemic and cellular copper metabolism. The body absorbs copper mostly through the small intestine, where it is then transported by blood to the liver for excretion into the bile. In tumor cells, interactions between several proteins maintain copper homeostasis. The entry and departure of copper ions into and out of the cell are controlled by the copper ion transporters SLC31A1 and ATP7B, whereas the transit of copper ions through the outer and inner mitochondrial membranes is controlled by COX17 and SLC25A3, respectively. Copper ions entering the cytoplasm and mitochondrial intermembrane space bind to GSH and MT or form copper-containing molecular chaperones such as SOD1 which is crucial for proper function of copper. To sustain normal cellular functions, COA6, SCO1 and SCO2 work together to mediate the transfer of copper to COX in the mitochondrial intermembrane space

Copper, a kind of indispensable transition metal, has two sides for cell. On the one hand, it served as co-factor for many enzymes by donating or receipting electronics [3], on the other hand, the accumulation of copper can lead to a series of cellular metabolic dysfunctions and eventually cell death [18]. People mainly obtain copper from food, out of which organ meats and shellfish tend to be the richest food sources of copper, and the current recommended intake of copper for adults should be 0.8–2.4 mg/day to maintain systemic copper homeostasis [19] (Fig. 2). Copper uptake occurs mainly through the small intestine, the small intestine epithelium took up copper ions via copper transporter 1 (CTR1) or called solute carrier family 31 member 1(SLC31A1), the transporter encoding by *slc31a1* on the cell surface. The copper was transported to another side of epithelium through copper chaperone antioxidant 1 copper chaperone (ATOX1) and exported into the bloodstream through the action of ATPase copper transporting alpha (ATP7A) [20]. Copper ions are transported in the blood by binding to proteins rather than being free. About 75% of copper ions are bound to ceruloplasmin (CP) in the non-exchangeable form, about 25% of copper ions are bound to human serum albumin (HSA) in the exchangeable form and about 0.2% of copper ions are bound to Histidine [21, 22]. Copper ions were then transported through portal system to the liver [23], which is the main organ for copper repository and also the main organ for copper excretion in the body. The copper storage function was believed to be mediated by metallothionein1/2 (MT1/2), two thiol-rich proteins, which bind copper ions in a pH-dependent manner through their cystine residues, however, their specific ability to bind and transfer copper is still unknown. Through the function of ATPase copper transporting beta (ATP7B), excess copper is excreted into the bile and leaves the body [24].

The role of copper in tumor processes has also been of interest to researchers. A significant increase in serum copper ion levels in tumor patients compared to normal patients has been observed in studies of lung cancer [25], prostate cancer [26], breast cancer [27], carcinoma of gallbladder [28], stomach cancer [29] and thyroid cancers [30]. Higher levels of copper ions were also observed in the gallbladder tissue of patients with gallbladder cancer [28]. Further, among lung cancer patients, those with worse clinical stage had higher serum copper ion concentrations and higher serum copper ion concentrations were also associated with worse clinical prognosis. The normal function of copper ions in cancer cells is dependent on the regulation of copper homeostasis and the interaction of different types of proteins (Fig. 2). The first group is proteins related to copper transport across the membrane. Consistent with small intestinal epithelial cells, the uptake of copper ions by tumor cells also requires the involvement of CTR1, and the elevated and decreased expression levels of SLC31A1 directly affect intracellular copper ion levels [31]. CTR1 mainly transports monovalent copper ions. After being transported to the cell surface in the blood, divalent copper ions are reduced to monovalent copper ions catalyzed by steap proteins [32], which are bound and maintained in the reduced state by two His-Met-Asp clusters at the nitrogen terminus of CTR [33], and thus transported into the cell. Cu was transported from the intermembrane space of mitochondria across the inner membrane into the mitochondrial matrix by the transmembrane transport protein solute carrier family 25 member 3 (SLC25A3) [34]. However how copper ions enter the intermembrane space of mitochondria through the outer membrane is still unknown. ATPases, including ATP7A and ATP7B, associated with the extracellular excretion of copper, export Cu ions bound to metal binding sites in the presence of ATP [35]. These proteins that mediate the transmembrane transportion of copper ions regulate their intracellular distribution. The second group is proteins that bind and store copper ions. MT and glutathione(GSH) served as naturally intracellular copper ion chelators, binding copper and thus preventing it from causing cell damage [36]. The third group is copper ion chaperones [37], interaction of which ensures proper copper cellular function. Cytoplasmic copper ion chaperones ATOX1 bind Cu(I) via two cysteine residues and transport it to the metal binding sites of ATP7B for further exportation. Copper chaperone for superoxide dismutase(CCS) directly interacted with and transported copper ions to superoxide dismutase 1(SOD1) [38]. With the involvement of O2, CCS can accelerate the disulfide formation of SOD1, which is essential for the correct spatial conformation and the enzyme activity [39]. SOD1 plays a role in catalyzing the generation of H2O2 from superoxide radicals and plays a key role in maintaining intracellular reactive oxygen species(ROS) homeostasis, and the inactivation of which can lead to the onset of cell death [40]. In addition to this there are a series of intra-mitochondrial copper ion chaperones that play an important role in the function of cytochrome c oxidase (COX), an important component of oxidative phosphorylation. These chaperones are involved in the composition and function of COX by storing or delivering copper ions [41]. Cytochrome c oxidase copper chaperone(COX17) carried copper ions from the cytoplasm into the intermembrane space of mitochondria [42], and further delivered copper ions to the cysteine residues of SCO1 with which it formed disulfide bonds [43]. Cytochrome c oxidase assembly factor 6(COA6) served as a thiol-disulfide oxidoreductase to reduce the formation of disulfide bonds between cysteine residues in synthesis of cytochrome c oxidase1/2 (SCO1/2) and substances other than copper [44], thus allowing copper binding [45]. The deficiency of COA6 lead to disorder of respiratory complex IV biogenesis [46]. In the effect of COA6, SCO1 and SCO2 transferred the copper ions obtained from COX17 to COX and participate in cytochrome c oxidase assembly [47]. Moreover, SCO1 and SCO2 were also involved in the regulation of cellular copper homeostasis, and the absence of both decreases cellular copper ion levels [48]. The maintenance of intracellular homeostasis of copper is dependent on the interaction of these four types proteins, and dysregulation of copper homeostasis will lead to disruption of cellular metabolism and even cell death.

### Cuproptosis and cancer signaling pathways

**Fig. 3:**
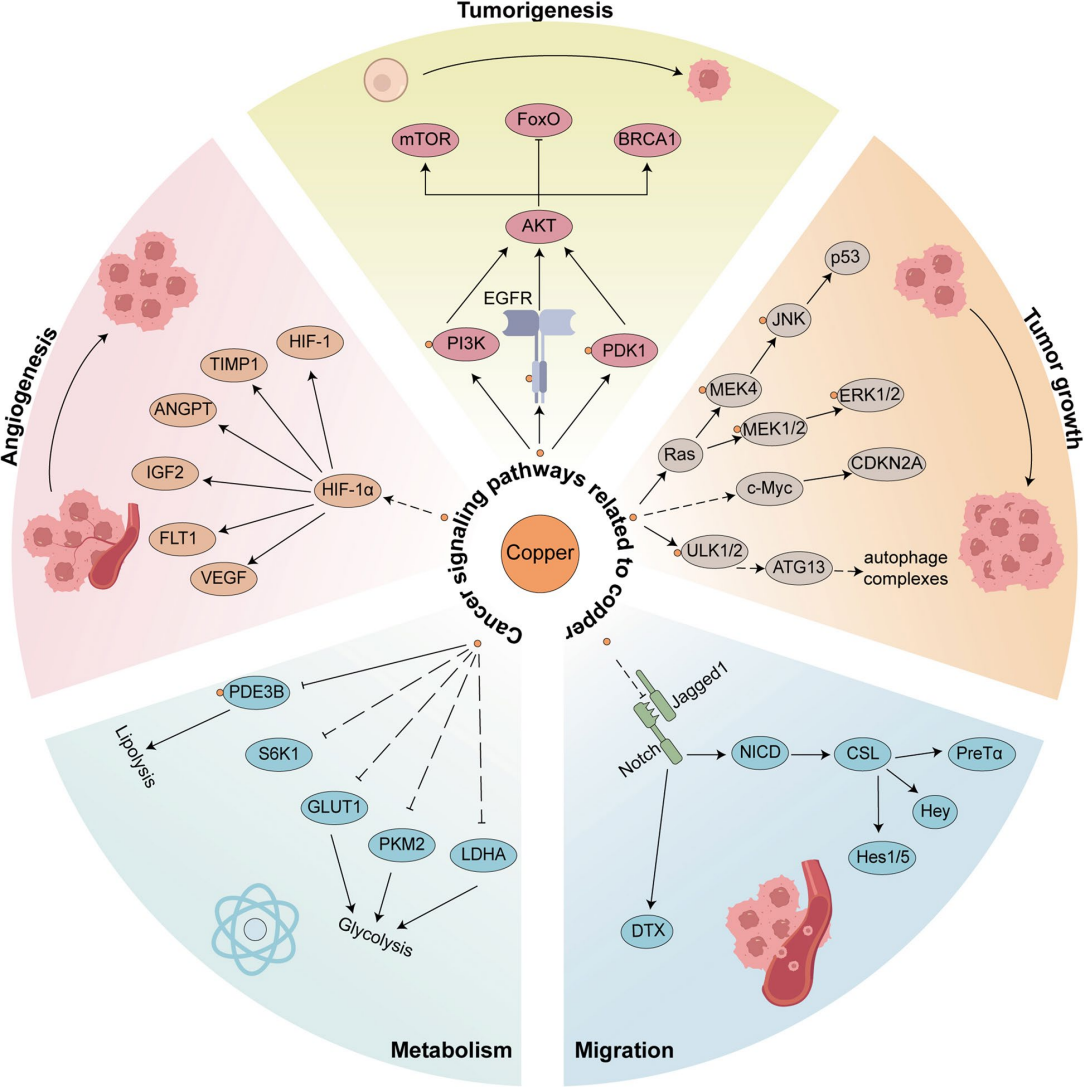
Copper and cancer singnaling pathways. Copper is strongly associated with the process of cancer and impacts them in a direct or indirect ways. Copper directly binds or activates EGFR, PDK1 or PI3K to promotes tumorigenesis. Copper also influences MAPK and autophagic pathways or indirectly changes c-Myc stability to influence tumor growth. Copper ions indirectly promotes HIFα or indirectly inhibits the Notch pathway ligand Jagged1 thus promoting vascular neoplastic migration. In addition, copper can also regulate PDE3B or S6K1 and thus modulates tumor metabolism

Copper is thought to be directly related to multiple signaling pathways in tumor cells, by binding and activating key molecules in multiple signaling pathways (Fig. 3). Copper were considered to play a critical role in receptor tyrosine kinase-related signaling pathways, which can bind and phosphorylate receptor tyrosine kinase(RTK) with no ligand binding and further lead to RKT activation. Activated RTK subsequently lead to phosphorylation of downstream extracellular regulated protein kinases(ERK) and agammaglobulinaemia tyrosine kinase(ATK), ultimately lead to cell migration and proliferation [49]. Copper ions are also thought to cause downstream activation by acting on different molecules of the phosphoinositide-3-kinase (PI3K)-AKT signaling pathway. On the one hand, Copper can directly activate the PI3K which leads to downstream AKT activation [50]. On the other hand copper bind to the histidine117 and histidine203 sites of pyruvate dehydrogenase kinase 1(PDK1) which lead to activation of AKT [51]. The AKT activation triggered by copper can further catalyze the phosphorylation and subcellular redistribution of forkhead box O1a(FoxO1a) and forkhead box O4(FoxO4), which promoted the cancer cell proliferation and tumor growth [52]. Activation of mitogen-activated protein kinase(MAPK) signaling pathway is also dependent on the presence of copper ions [53], Copper can directly bind to mitogen-activated proteinkinase kinase 1(MEK1) to promote the phosphorylation of ERK1/2 and further activate the downstream c-Jun N-terminal kinase(JNK) to regulate tumor growth [54].

The autophagy pathway can recycle metabolic waste from tumor cells to ensure their energy needs or allow them to escape apoptosis, ultimately leading to proliferation of tumor cells. Copper directly binds Unc-51 Like autophagy activating kinase(ULK) and acts as its regulator to promote phosphorylation and activation of autophagy related 13(ATG13), resulting in the formation of the autophagic complex and ultimately tumor growth [55, 56]. Consistent with the MAPK signaling pathway, autophagic pathway can also promote cancer cell survival and is directly affected by copper ions. In B-Raf proto-oncogene(BRAF)-driven lung adenocarcinoma cells, loss of CTR1 leads to a decrease in copper ion concentration that is directly associated with reduced function of MEK1/2 and ULK1/2, key kinases in both signaling pathways [57]. In addition to interacting directly with important proteins in the pathway, copper ions can also affect them indirectly to regulate the biological behavior of cancers. The Notch pathway is often considered as a tumor suppressor, which is extensively involved in the development of malignant tumours [58]. Copper ions promote shedding of the notch ligand Jagged1 on cell surface and promote tumor cell migration [59]. Numerous reports have revealed that copper ions have a close relationship with tumor angiogenesis [60], which was dependent on the interaction between copper and hypoxia inducible factor 1 subunit alpha(HIF-1α)-related signaling pathways. Copper mediates HIF-1α binding to the critical motifs of target gene promoters through a CCS-dependent manner thereby upregulating the expression of affected genes such as hypoxia inducible factor 1(HIF-1) [61, 62]. Even under normoxic conditions, copper can directly increase the stability of HIF-1α, which in turn promotes the expression of target genes such as vascular endothelial growth factor(VEGF), leading to tumor angiogenesis [63]. Previous research has also emphasized the significance of copper in inflammation promoting effects by interacting with NFκB pathways [64]. Inflammatory cytokines promote elevated intracellular copper levels and lead to X-linked inhibitor of apoptosis(XIAP) activation, which promotes NFκB activation and tumorigenesis. A strong relationship between copper and lipolysis pathways has been reported in the previous research. The Wnt signaling pathway maintains the renewal balance of human cells, and activation of whose genes exerts a pro-tumor effect in tumor cells [65]. A much-debated question is whether copper up or down regulated the level of C-myc. The previous study has found that increasing intracellular copper ion concentration through disulfiram(DSF) leads to a decrease in the expression of β-catenin and C-myc, two important molecules of the Wnt pathway, thereby inhibiting tumor growth [66]. However, a more recent research argued that the Copper elevates C-myc stability by promoting phosphorylation at its threonine 58 and serine 62 sites [67]. The level of tumor cell metabolism also has an impact on the biological behavior of tumors, and copper ions can regulate tumor metabolism through interactions with related molecules within the lipid or sugar metabolic pathways. Copper has been shown to regulate lipolysis through interaction with cysteine residues of phosphodiesterase 3B(PDE3B), the phosphodiesterase that degrades cAMP [68]. Moreover, Cu is also thought to inhibit the expression of S6K1 and its downstream glycolysis-related molecules, including GLUT1, PKM2 and LDHA, thereby suppressing tumor growth [69]. Collectively, copper plays a direct or indirect role in cancer signaling pathways and cancer properties, which further emphasizes its importance in cancers.

### From copper induced cell death to cuproptosis

During the past few years, the link between copper and programmed cell death has long been at the center of much attention and the mechanism of copper induced cell death has long been researched. Copper has been known to cause cell death in the 1980s [70], however, the exact mechanism has not been elucidated. Copper ionophores, a lipid-soluble molecule that binds copper ions reversibly, played an important role in the discovery of cuproptosis, and may be involved in clinical treatment as antitumor agents [71]. Copper ionophores may transport copper ions through the plasma membrane or mitochondrial membrane structure of a cell. DSF, a drug that has been used to treat alcohol dependence, also functions as a copper ionophore and is thought to cause cell death [72], in the same way that elesclomol(ES), another copper ionophores, was also believed to have the ability to kill cells [73]. In studies on copper ionophores ES and DSF, many researchers have investigated the mechanism by which these copper ionophores cause cell death, suggesting that such cell death was caused by copper rather than copper ionophores, however, the exact mechanism was not indicated [74]. ROS is mainly derived from intracellular redox reactions in which mitochondria play a key role [75], which is also consistent with the correlation between copper induced cell death and mitochondrial metabolism. Among the studies on ES-induced cell death, researchers generally agree that the cell death caused by ES is mediated by elevated levels of ROS due to various mitochondrial-related factors. A study in 2012 on melanoma cell lines concluded that ES transported copper and led to reduced levels of mitochondria related proteins, thereby lead to increase of ROS and further inhibition of tumor cell proliferation [74]. In a 2013 study conducted in Human leukemia K562 cells, copper ions were suggested to be able to oxidize ascorbic acid and react with H2O2 to produce more damaging ROS after entering cells via ES transport [76]. It was suggested in the study on ES in 2015 that ES-Cu may have multiple roles, including blocking cells in the G1 phase, damaging DNA and affecting mitochondrial membrane potential [16]. A 2016 study on AT-rich interaction domain 1A(ARID1A) concluded that ES can act on the mitochondrial respiratory chain and lead to increased levels of intracellular reactive oxygen species and cell death through a ROS-mediated mechanism [77]. Other studies on tumor targeting by copper ionophores have proposed the same mechanism as previously described [78, 79]. However, in 2019 Tsvetkov et al. found that Hi-Mito condition (a rise in mitochondrial respiration, which can be induced by replacing Glucose with Galactose) leads to a resistance to PI but together with a higher vulnerability to ES, the copper ionophore. Ferredoxin 1(FDX1) was identified as the gene most associated with ES sensitivity, which was directly bound by ES-Cu and lead to inhibition of the iron-sulfur cluster (Fe-S cluster) formation function [14]. In 2021, studies in glioblastoma stem like cells revealed that ES-Cu can act directly on the mitochondrial membrane and lead to changes in the mitochondrial membrane potential, with low concentrations of ES leading to hyperpolarization and high concentrations of ES leading to depolarization, and this effect on the mitochondrial membrane potential can be inhibited by tetrathiomolybdate(TTM) [7]. In common with ES, DSF can also transport copper into the cell, and copper is thought to act on the mitochondrial respiratory chain, leading to elevated levels of ROS [17]. In addition to causing elevated levels of ROS, the interaction of DSF-Cu with Npl4 was also thought to be closely associated with copper-induced cell death. Copper is thought to inhibit the ubiquitinated protein degradation function of p97 by interacting with Npl4, possibly leading to its aggregation [80, 81] or directly binding and inhibiting its conformational transition [82], ultimately leading to cell death. In summary, in past studies on Cu-induced cell death, most researchers have attributed this cell death to the action of copper on the mitochondria resulting in the production of ROS. However, in studies on the mechanism of action of ES, the cytotoxic effect caused by ES-Cu was not eliminated by using 5 mM ROS inhibitor N-Acetylcysteine (NAC), and the cytotoxic effect was only partially eliminated by 10 mM NAC, so ROS-mediated cell death may not be the main mode of cell death caused by copper. The exact form of cell death caused by copper ions has been controversial, and in the past researchers have mostly regarded it as apoptosis [83–86], autophage [87, 88] or ferroptosis [89, 90], which can be proved by the evidence of cell viability assay, western blotting, flow cytometry and immunofluorescence staining. Until March 2022, Tsvetkov et al. identified the mechanism of copper induced cell death, which was named cuproptosis, researches on the mechanism of copper induced cell death have reached a new milestone. None of the inhibitors for apoptosis, necroptosis, ROS induced cell death or ferroptosis but the copper chelator can rescue cells from copper induced cell death [4], and the expression level of cleaved caspases were not increased. In other words, among the aforementioned evidence, the evidence of cell viability assay and western blotting have been disproved, while the others remain to be further verified.

The cell death triggered by ES decreased significantly by reducing copper binding function and completely disappeared after removal of this function. ES that does not carry copper ions cannot independently cause cell death [14]. Except for ES and DSF, other ionophores can also lead to same cellular effects. GSH was a natural intracellular chelator of copper ions, reduction of which can also lead to an increase in intracellular copper concentrations and ultimately lead to the cell death [4]. Moreover, in a WD-related model, it was found that breakdown of copper homeostasis through downregulation of ATP7B could also lead to the development of cell death [91].In summary, cell death caused by copper ionophores is a type of cell death that is triggered by copper independently of the known modes of cell death.

**Fig. 4:**
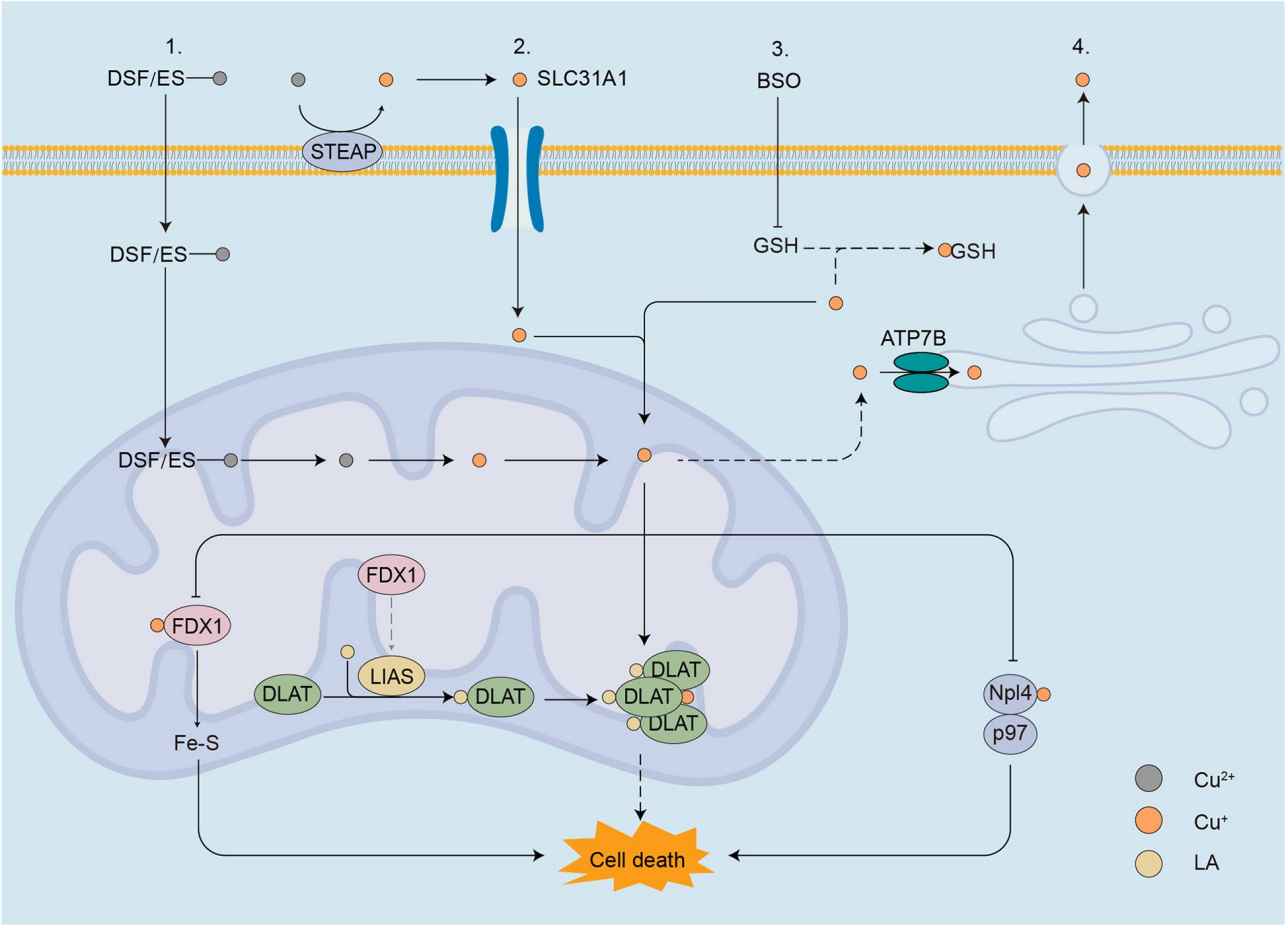
Schematic of cuproptosis mechanism. Cuproptosis can be triggered by elevating intracellular free copper ion concentration in four ways involved in the absorption, export and storage of copper: (1) treatment with copper ionophores, which shuttle copper into the cell directly, such as ES and DSF; (2) overexpression of SLC31A1, the copper permease specific for reduced copper ion; (3) inhibition of glutathione (GSH) synthesis through BSO, without which free copper ion was released; (4) knockdown of ATP7B, decreasing copper export. Excessive Cu(I) binds to lipoyled DLAT and further leads to DLAT oligomerization, which, together with copper-induced reduction of Fe-S stability or inactivation of Npl4-p97, can lead to the onset of copper-induced cell death. ES elesclomol, DSF disulfiram, BSO L-Buthionine-sulfoximine

Cuproptosis was thought to be interact with components of the TCA cycle in mitochondria and involves a conserved post-transcriptional protein modification pathway, lipoylation [4], the mechanism of which displayed in the form of a schematic (Fig. 4). To identify the exact component of mitochondrial respiration that interacted with copper, Oligomycin (ATPase inhibitor), FCCP (uncoupler) and antimycin A/rotenone (electron transport chain inhibitor) has been used to measure the oxygen consumption rate (OCR). The OCR showed significantly reduced spare capacity of respiration rather than basal respiration or ATP-linked respiration, which proved that copper directly interact with the TCA cycle rather than neither the electron transport chain (ETC) complex nor the ATP production component [4]. Through genome wide knockout screens, metabolism screens and individual gene knockout studies, CKGscrucial for cuproptosis has been discovered and validated (Table 1), knockout of seven of which could lead to the rescue of copper ionophores [4]. These seven genes can be divided into three groups, FDX1, LA pathway-related genes (LIAS and LIPT1) and genes encoding components of pyruvate dehydrogenase complex (PDC) which play a crucial role in mitochondrial respiration (DLAT, DLD, PDHA1 and PDHB), all of which were correlated with LA pathway. FDX1 was considered may act as the upstream of LA pathway, and under the regulation of FDX1, LIAS linked lipoyl moiety to DLAT, which was essential for the function of mitochondrial PDC. Cu(I) directly bound with lipoyl moiety of lipoylated DLAT through disulfide bond and further lead to DLAT oligomerization and further proteotoxic stress and finally result in cell death [4].

**Table 1:** Functions and clinical values of cuproptosis validated genes

Gene	Full name	Subcellular locations	Functions	Role in cuproptosis	Clinical values	Ref
FDX1	Ferredoxin 1	Mitochondrion matrix	Fe-S cluster biosynthesis ruduce Cu(II) to Cu(I) synthesis of various steroid hormones electron transport intermediate for mitochondrial cytochromes P450	act as the upstream of LA pathway ruduce Cu(II) to Cu(I)	FDX1 high expression correlaed with better prognosis in many cancers	[14, 91–99]
LIAS	Lipoic Acid Synthetase	Mitochondrion	converting the octanoylated domains into lipoylated derivatives	Regulated by FDX1, and involved in lipoylation pathway	LIAS high expression led to worse OS and FP, and correlated with more advanced lung cancer staging	[100, 101]
LIPT1	Lipoyltransferase 1	Mitochondrion	Catalyzes the transfer of the lipoyl group from lipoyl-AMP to the specific lysine residue of lipoyl domains of lipoate-dependent enzymes	Regulated by FDX1, and involved in lipoylation of DLAT	LIPT1 high expression led to better prognosis	[102–104]
DLD	Dihydrolipoamide Dehydrogenase	mitochondrion and nucleus	E3 component of the pyruvate dehydrogenase complex	not montioned	NA	[105, 106]
DLAT	Dihydrolipoamide S-Acetyltransferase	Mitochondrion matrix	component of pyruvate dehydrogenase complex, mediate the conversion of pyruvate to acetyl-CoA	under the influence of copper, lipoylated DLAT oligomerization lead to cell death	NA	[107]
PDHA1	Pyruvate Dehydrogenase E1 Subunit Alpha 1	Mitochondrion matrix	component of pyruvate dehydrogenase complex, mediate the conversion of pyruvate to acetyl-CoA	not montioned	PDHA1 high expression led to worse OS and FP in LUAD and low expression correlaed with HCC	[108]
PDHB	Pyruvate Dehydrogenase E1 Subunit Beta	Mitochondrion matrix	component of pyruvate dehydrogenase complex, mediate the conversion of pyruvate to acetyl-CoA	not montioned	NA	NA
MTF1	Metal Regulatory Transcription Factor 1	Nucleus and Cytoplasm	Zinc-dependent transcriptional regulator for metal ions adaption	knock out lead to sensitive of cuproptosis	NA	[109]
GLS	Glutaminase	Mitochondrion. Cytoplasm and cytosol	Catalyzes the catabolism of glutamine	knock out lead to sensitive of cuproptosis	GLS high expression led to worse OS	[110, 111]
CDKN2A	Cyclin Dependent Kinase Inhibitor 2A	Nucleus and Cytosol	inducing cell cycle arrest in G1 and G2 phases	knock out lead to sensitive of cuproptosis	CDKN2A higher expression led to risk in LUAD but protective function in BRCA	[112–114]
SLC31A1	Solute Carrier Family 31 Member 1	Cell membrane	High-affinity, saturable copper transporter involved in dietary copper uptake	overactivaiton lead to intracellular copper accumulation	SLC31A1 alleles were associated with a worse prognosis in LUAD and BRCA	[112–120]
ATP7A	ATPase Copper Transporting Alpha	Cell membrane, trans-Golgi network membrane, plasma membrane	ATP-driven copper ion pump	knock out lead to intracellular copper accumulation	NA	[119]
ATP7B	ATPase Copper Transporting Beta	Cell membrane, trans-Golgi network and membrane	ATP-driven copper ion pump	knock out lead to intracellular copper accumulation	ATP7B alleles were associated with a decreased risk of LUAD	[99, 118]

Although Tsvetkov et al. used concise cell lines and mouse models to elaborate part of the mechanism of copper action in cuproptosis, there are still some unanswered questions about cuproptosis that need further study. To begin with, the characteristic manifestations of cuproptosis have not been described. On the one hand, the cellular morphological changes of cuproptosis have not been described, whether cuproptosis occurs with characteristic or sequential morphological manifestations, and on the other hand, the characteristic changes that occur at the molecular or cellular level after the induction of cuproptosis have not been identified, thus lacking effective means to assess whether cuproptosis has occurred [4]. Furthermore, the downstream pathways of DLAT oligomers, which play an important role in cuproptosis, have not been described. In previous studies, only the toxic effects of DLAT oligomerization were described and it was assumed that this DLAT oligomerization leads to proteotoxic stress and ultimately to cell death. However, the direct mechanism between DLAT oligomerization and cell death has not been determined [4]. In addition, in studies on copper ionophores, either ES-Cu or DSF-Cu has been suggested to lead to increased proteotoxic stress in other ways besides DLAT oligomerization. For example, ES-Cu can lead to decreased stability of Fe-S through FDX1 [14] and DSF-Cu can affect the cellular ubiquitination degradation pathway by interaction with Npl4 [80–82], further leading to increased proteotoxic stress and ultimately leading to cell death. However, whether there are interactions between these events leading to increased cellular proteotoxic stress remains to be investigated. Finally, the function of FDX1 has not been sufficiently mentioned, FDX1 is thought to play a central role in the process of cuproptosis and was considered to act as an upstream of the LA pathway, but whether its function is achieved through direct interaction with LIAS or the action of some other proteins needs further investigation [4].

### Validated cuproptosis key genes: functions and clinical values

Ten genes were identified by Tsvetkov et al. through whole genome knockout, deletion of which could lead to an altered risk of cuproptosis. While the analysis about the copper homeostasis suggested that SLC31A1, ATP7A and ATP7B could also affect cuproptosis by regulating the intracellular copper ion concentration [4]. Therefore these 13 genes might play an important role in the mechanism of cuproptosis and were considered as CKGs. The function and subcellular location of these CKGs are listed in Table 1. These CKGs have important cellular roles and are often closely related to energy metabolism and metal homeostasis processes. As the concept of cuproptosis was introduced, a considerable number of researchers focused on the role played by these CKGs in different tumors, indicating the expression levels and clinical significance of CKGs in different cancers.

FDX1 encodes Ferredoxin1, the iron–sulfur protein that was involved in multiple redox reactions. FDX1 served as monooxygenase of cytochrome P450 and was involved in steroidogenesis [92]. Also, it has proved that FDX1 was crucial for the iron–sulfur cluster biogenesis [93, 94]. In addition, FDX1 was also considered can reduce Copper ion from divalent to monovalent and exhibited stronger cytotoxic properties and cellular function [14]. In the process of cuproptosis, FDX1 played a central role which regulated the process of protein lipoylation as the upstream of LA pathway [4]. Compared to paired normal tissues, FDX1 expression levels were upregulated in glioblastoma (GBM) and female genital tumors, and downregulated in solid tumors like lung adenocarcinoma (LUAD) and hepatocellular carcinoma (HCC) [95]. High levels of FDX1 were considered to be associated with poor prognosis in patients with head and neck squamous cell carcinoma (HNSC) and low grade glioma (LGG). In contrast, higher FDX1 expression lead to better prognosis in patients with cervical squamous cell carcinoma(CESC) and clear cell renal cell carcinoma (KIRC) [95–97]. In KIRC and colorectal cancer (CRC),patients with lower expression level of FDX1 was condisered to suffer more advanced and metastatic cancers together with poor overall survival(OS) and disease free survival(DFS) [98, 121].

Lipoic acid is a crucial substance which is required for enzymes involved in oxidative decarboxylation of mitochondrial metabolism intermediates [122]. Protein expressed by LIPT1, LIAS all belong to the LA pathway, which mediated the post-transcriptional lipoic modification of proteins like PDC [122], was both crucial for cell normal cellular activity and cuproptosis. LIAS synthesized lipoic acid by introducing two sulfhydryl groups at the C6 and C8 sites of the octanoic acid moiety, deficiency of which led to neonatal epilepsy, disorder in mitochondrial energetic metabolism and elevated glycine [100]. Similarly, LIAS expression levels tend to correlate with prognosis in different patients, high LIAS expression in lung cancer was suggested to have a poor prognosis, while in KIRC and ovarian cancer it was considered to be associated with a better prognosis [101]. LIPT1catalyzed the transfer of a lipoyl group to the lysine residue of the target enzymes [102], deficiency of which will lead to Leigh disease together with a deficiency of PDC [103].In addition, cancer cells growth and invasion were prevented by knocking down the expression of the LIPT1 gene [104].

DLAT, DLD, PDHA1 and PDHB served as three vital subunits of PDC [123], which is the important component of the mitochondrial aerobic respiration process [124] and played a key role in the cuproptosis process [4], and the expression levels of which were also believed to be related to tumor prognosis. PDHA1 and PDHB form the α1 and β subunits of the PDC E1 component, respectively, and together form pyruvate dehydrogenase mediating pyruvate decarboxylation [125]. High levels of PDHA1 were thought to be associated with a better prognosis for lung cancer patients and can be used as a biomarker for the tumor microenvironment [108]. DLAT form the E2 component of PDC that acts as a dihydrolipoamide acetyltransferase catalyzing the biosynthesis of acetyl coenzyme A(acetyl-CoA) [107]. Lipoylated DLAT played an important role in the process of cuproptosis, where its oligomerization in the presence of copper leads to proteotoxic stress and ultimately to cell death. While DLD participated in the formation of the E3 component of PDC as dihydrolipoamide dehydrogenase catalyzes the formation of NADH [105, 106]. PDC generates acetyl-CoA and NADPH through cooperative interactions between three subunits, which is the control step for the mitochondrial oxidative phosphorylation [126], and its phosphorylation mediated by PDC kinase leads to inactivation [127].

CDKN2A, GLS and MTF1 have also been demonstrated that related to the cell cuproptosis sensitivity [4]. GLS mainly catalyzes the catabolism of glutamine, which convert glutamine into glutamate, and is also involved in the maintenance of glutamate homeostasis. Dysfunction of GLS can lead to glutamine overload affecting the physiology and structure of the central nervous system [110], and its hyperactivity can lead to glutamate overload also leading to neurodevelopmental delays [111]. MTF1 served as a transcriptional regulator of cellular adaptation to heavy metals, which activates the transcription of copper binding protein MT, by binding to the metal response element in the promoter of MT [109]. GLS and MTF-1 may influence the sensitivity of cells to cuproptosis by affecting the intracellular levels of the copper ion-binding substances GSH and MT. CDKN2A was suggested to interact with and inhibit cyclin‑dependent kinase inducing cell cycle arrest in G1 phases. In the past, studies have focused on the tumor suppressor gene role of CDKN2A, and mutations of which led to loss of growth control in breast cancer(BRCA) [112], HNSC [113] and ovarian cancer cells [114], but its role in cuproptosis remains to be further investigated. CDKN2A was suggested to be highly expressed in BRCA and LUAD, and in addition, high CDKN2A expression was thought to correlate with immune cell infiltration levels [116, 117].

Dysregulation of aforementioned copper homeostasis maintainer SLC31A1, ATP7A and ATP7B also leads to disruption of cellular functions [3]. As previously described, SLC31A1 mediates copper entry into cells while ATP7A and ATP7B mediate copper exit from cells, all functioning as copper carriers that closely related to the shuttle of copper [115, 118, 119]. High expression of SLC31A1 has been considered to be associated with poor clinical outcome of BRCA patients by two independent researches [120, 128]. Researchers also found that the microsatellites of SLC31A1 and ATP7B were associated with lung cancer risk, suggesting that the expression levels of copper homeostasis-related genes may also influence the process of lung cancer development [99]. Previous research on these genes has uncovered the ways in which they may influence or be influenced by cuproptosis, as well as the potential significance of their involvement in the connection between cuproptosis and cancers. However, further studies are needed to explore and validate these functions.

### Cuproptosis related genes and cancer prognosis

There is a growing interest in cuproptosis since demonstration and most investigators made exploration on the relationship between cuproptosis and cancers. Researchers have used online databases to analyze some of the numerous genes that may play an important role in the link between cuproptosis and cancer, and based on which to predict the cancer features and prognosis of patients. As shown in Table 2, prognostic signatures constructed by CRGs and their clinical values have been listed.

**Table 2:** Association of cuproptosis related genes and cancers

Ref	database	cancer	risk factor	protective factor	microenviroment condition	Clinical value
[129]	TCGA	LUAD	DLAT, DLD, DLST and PDHA1	DBT and LIPT1	high risk related to immune cell infiltration reduction and less activity in HLA, type I and II IFN response	high risk led to worse OS together with higher mortality and more advanced T and N stage
[130]	TCGA, GSE68465	LUAD	DLAT	CDKN2A, PDHA1and MTF1	high risk led to less immune cell infiltration	higher risk score corelated with worse OS, more advanced pathological stage, positive lymph nodes and more severe tumor status
[131]	TCGA, GTEx, NODE, ICGC	HCC	ATP7A, LIPT1, DLAT, MTF1, GLS, and CDKN2A	none	high risk related to immunosuppressive microenviroment, immune cell infiltration reduction and macrophage polarization inhibition	high risk score correlated with worse survival
[132]	TCGA	liver cancer	DLAT	ATP7A, GLS	higher risk score correlated with worse immune status and higher expression of immune check point genes	higher risk correlated with worse OS and clinicopathological features
[133]	TCGA, GSE76427	HCC	MANEA, PGM2, PTTG1	CGNL1, ALAS1	higher risk score correlated with actived CD4 + T	higher risk score correlated with higher mortality and worse OS
[134]	TCGA, ICGC, TISCHGSE64041, GSE14520/GPL3921, GSE76427, GSE104580, GSE109211, and GSE25097	HCC	CAT, SLC27A, EHHADH, ALDH5A1	none	higher risk score correlated with protumor immune infiltration and higher expression of immun check point genes	higher risk score correlated with worse OS, PFS and clinicopathological features
[135]	TCGA, GSE76427	HCC	PBK, MMP1, GNAZ, GPC1	AKR1D1	higher risk score correlated with protumor immune infiltration	higher risk score correlated with worse OS
[136]	TCGA, GSE14520	HCC	KIF2C, PTTG1, CENPM, CDC20, SFN	CYP2C9, CFHR3	higher risk score correlated with less immune cell infiltration and higher expression of immun check point genes	higher risk score correlated with worse OS
[137]	TCGA, GTEx	CC	ATP7A, DLAT, GCSH	DBT, FDX1, LIPT1, PDHA1	higher risk score correlated with less immune cell infiltration	higher risk score correlated with worse OS and more advanced clinical stage
[138]	TCGA	USEC	CDKN2A, GLS, and LIPT1	none	not mentioned	higher risk correlated with worse OS, PFS and DFS
[139]	TCGA, GSE22138	UVM	LIPT1, DLD, PDHA1, CDKN2A	LIAS, PDHB, MTF1, GLS	no significant difference in the total immune cell infiltration	higher risk correlated with more advanced clinical stage
[140]	TCGA, GTEx, CGGA	LGG	C21orf62, DRAXIN, ITPRID2, MAP3K1, and MOXD1	none	higher risk score correlated with higher TME scores and lower CSC index, also with higher expression of immun check point genes	higher risk correlated with worse OS and more cases of death
[141]	TCGA, GTEx, CGGA311, CGGA668, GSE108474, GSE13041, GSE16011, GSE43289, GSE43378, GSE4412, GSE4412, GSE68838, and GSE83300	giloma	H19, CYTOR, IGFBP2 and CHI3L1	KLRC2 and C5orf38	higher risk score correlated with higher immune infiltration and less immune function	higher risk correlated with more advanced clinical stage
[142]	TCGA, CGG, AGSE84465	giloma	SLC31A1, NFE2L2, MT1M, MT1H, MAP1LC3A, FDX1, COX19, ARF1, AOC1	LIAS, CYP1A1, ATP7B	higher risk score correlated with higher immune infiltration and less immune function	higher risk correlated with more advanced clinical stage
[143]	TCGA, CGGA	giloma	FDX1, DLD, MTF1	DLAT, CDKN2A	higher risk score correlated with higher protumor immune infiltration	higher risk score correlated with worse OS
[144]	TCGA, GSE17538, GSE29623, GSE39582	CRC	CDKN2A, GLS	DLAT	higher risk score correlated with less immune cell infiltration and more stormal cells	higher risk correlated with more advanced clinical stage and worse OS
[145]	TCGA, GSE61304	BC	PGK1, PRDX1, MAL2, and SURF4	RPL14, PSME1	higher risk score correlated with higher immune activation	higher risk correlated with worse OS and PFS
[146]	TCGA, GSE58812, GSE135565 and GSE65194	TNBC	ATP7A	LIPT1	higher risk score correlated with less immune cell infiltration and lower TME score	higher risk correlated with worse OS and more advanced clinical stage
[147]	TCGA, GSE168410, GSE20685 and GSE20711	BC	DLAT, SNX3, TTC3, PHF20, RTN4, SURF4, SDC1, KDELR2, BAMBI, ANXA5, MARVELD	RBP1, TPT1, MDK, RPLP1, ETV6	higher risk score correlated with more active immune function and higher immune check point genes expression	higher risk correlated with worse prognosis
[148]	TCGA, GSE40435 and GSE53757	KIRC	CDKN2A	FDX1, DLAT	not mentioned	higher risk correlated with worse OS and PFS
[149]	TCGA, E-MTAB-1980, ICGC, GSE64052	KIRC	FDX1, CDC42BPG, C11orf52, GNG7, PAQR5, ENAM, WDR72, SDR42E1, BSPRY and KDF1	TMEM214, CCM2 and P3H4	higher risk score correlated with higher expression of immune check point genes	higher risk correlated with worse OS and DFS
[150]	TCGA, GSE12606, GSE53000, and GSE53757	KIRC	none	ENAM, WDR72, CLDN10, HMGCS2, CYFIP2, QRFPR	higher risk score correlated with more immune cells infiltration	higher risk correlated with worse clinicopathological features
[151]	TCGA	HNSCC	PRKN, MT1E, CXCL8, COX11, COX5A, COX19, ACLY	CYP2D6, ABCB1, CCL5, LOXL1, CDKN2A, BCL2, DAPK2	higher risk score correlated with less immune cell infiltration and immune function	higher risk correlated with worse OS and clinicopathological features
[152]	TCGA	HNSCC	not mentioned	not mentioned	higher risk score correlated with higher expression of immune check point genes, less immune function and lower TME score	higher risk correlated with worse OS, PFS, DFS and clinicopathological features
[153]	TCGA, GSE41613 and GSE65858	HNSCC	PRELID2, ANP32B, MRPL47, and CCDC59	CDKN2A, WDR90, NLRX1 and KCNK6	higher risk score correlated with higher expression of immune check point genes	higher risk correlated with worse OS
[154]	TCGA, GSE41613 and GSE42743	HNSCC	not mentioned	not mentioned	higher risk score correlated with higher expression of immune check point genes	higher risk correlated with worse OS and more advanced clinical stage
[155]	TCGA	ESCA	SLC25A5, SLC23A2, PDHX, ATP7A, and COX7B	PHID2	higher risk score correlated with more immune cells infiltration	higher risk correlated with worse prognosis
[156]	TCGA, ICGC	PAAD	KRAS, TP53, BRCA1, BRCA2, DLAT	CDKN2A, SMAD4, LIAS, LIPT1, DLD, PDHA1, MTF1, GLS	higher risk score correlated with low TME scores and expression of immune check point genes	higher risk correlated with worse OS
[157]	TCGA, GTEx	PAAD	DLAT	LIPT, LIAS	higher risk score correlated with more immune cells infiltration	higher risk correlated with worse OS
[158]	TCGA, ICGC, GTEX, IMvigor210	PAAD	DLAT, TIMMDC1, GSS	NDUFB2, LIAS, NDUFA8, ISCA2	higher risk score correlated with more immune supression	higher risk correlated with less survival probablity
[159]	TCGA, GSE30219, GSE31210 and GSE37745	LUAD	LINC00205, LINC00592 and AL162632.3S	AC026355.2, LINC02848 and ZNF571AS1	higher risk score correlated with greater potential for immune escape and suppressed immune function	higher risk score correlated with worse OS, PFS and higher mortality
[160]	TCGA, GSE130740	LUAD	AL031667.3, AL606489.1 and MIR31HG	AC008764.2, AL022323.1, ELN-AS1 and LINC00578	higher risk score correlated with lower score of most pathways related to immune and less immune cells infiltration	higher risk correlated with worse OS
[161]	TCGA	HCC	miR-767-5p, miR-5003-3p, miR-137-3p, miR-760, miR-548f-3p, miR-3171, miR-3189-3p, miR-3620-3p, miR-3911, miR-4652-3p, miR-504-3p, miR-892a, miR-548aq-5p	miR-67645p	higher risk score correlated with higher expression of immuno check point genes	higher risk correlated with worse OS
[132]	TCGA	liver cancer	POLH-AS, AL117336.2, MKLN1-AS, AC005479.2, AL928654.1, AL031985.3	none	higher risk score correlated with worse immune status and higher expression of immune check point genes	higher risk correlated with worse OS, more advanced clinical stage
[162]	TCGA, GTEx	HCC	AC138904.1, DEPDC1-AS1, GIHCG, AC145343.1	AC099329.2, DNMBP-AS1	higher risk score correlated with actived CD4 + T and higher expression of immune check ponit genes	higher risk correlated with worse OS
[163]	TCGA	PHC	MIR210HG, AC099850.3, AL031985.3, C012073.1, MKLN1-AS, KDM4A-AS1 and PLBD1-AS1	none	risk score correlated with less immunce cell infiltration	higher risk correlated with worse OS
[164]	TCGA, GTEx	CC	AL354733.3 and AC009902.2	AL441992.1, LINC01305, AL354833.2, CNNM3-DT and SCAT2	risk score correlated with less immunce cell infiltration and down regulation of immune functions and less Immune checkpoint gene expression	higher risk correlated with worse OS and PFS and worse clinicalpathological features
[165]	TCGA, GSE16088	OS	AL645608.6, AL591767.1 and UNC5B-AS1	CARD8-AS1, AC098487.1 and AC005041.3	higher risk score correlated with less immune infiltration and immune function	higher risk correlated with worse OS
[166]	TARGET	OS	AL033384.2	AL031775.1, AC110995.1 and LINC00565	higher risk score correlated with higher infiltration of immunosuupressive cell and less immune effective cell infiltration and immune function	higher risk correlated with worse OS
[167]	TCGA	SKCM	none	VIM-AS1, AC012443.2, MALINC1, AL354696.2 and HSD11B1-AS1	higher risk score correlated with less immune infiltration, downregulation of immune function and immune check point gene expression	higher risk correlated with worse OS
[168]	TCGA	CM	AC009495.1	LINC01150, EBLN3P, MIR100HG, WAC − AS1, LINC00339 and USP30 − AS1	higher risk score correlated with less immune infiltration and less immune check point genes expression	higher risk correlated with worse OS
[169]	TCGA, GTEx, CGGA	LGG	CRNDE, FAM181A-AS1	HAR1A	higher risk score correlated with higher immune cells infiltration, TME scores and immune check point genes expression	higher risk correlated with worse clinicalpathological features, lower survival rate and worse clinical outcone
[170]	TCGA	CRC	AP003119.3, RNF216P1, AC156455.1, AL360270.1, AC139720.2, AC092614.1, LRP4-AS1, AP003555.1, AL513550.1 and AL512306.2	AC073896.3, LINC00511, AC026979.4, AC103703.1, LRP1-AS	higher risk score correlated with supressed immune function	higher risk correlated with worse OS and PFS
[171]	TCGA	CRC	AP001619.1, AC020917.2, AC002066.1, LINC01252, AC010789.2, LINC02542, AL356804.1 and ZFHX2-AS1	AC008752.2 and AC012313.5	higher risk score correlated with supressed immune function, less immune cell infiltration and less expression of immune check point genes	higher risk correlated with worse OS and clinical outcome
[172]	TCGA	CRC	LINC00861, AC090517.2, AC01233.5, AL513550.1, AC026979.4, AC064836.3, PRKAR1BAS2, LINC02175, ZNF775AS1, AL161729.4	AC073896.3	higher risk score correlated with less iimmune cells infiltration	higher risk correlated with worse clinical outcome
[173]	TCGA, GSE42743	OSCC	C6orf99, AC010894.2, AC099850.4, RPL23AP7	AC090587.2, AL513190.1, AC098484.2	higher risk score correlated with lower TME score	higher risk correlated with worse prognosis and higher mortality rate
[174]	TCGA	BC	AC079922.2, ZNF197-AS1, AC002398.1, AL451085.3, LINC02446	GORAB-AS1, AL589765.4, AC005696.4, CYTOR, YTHDF3-AS1, AC008771.1	higher risk score correlated with inactive immune function and less expression of immune check point genes	higher risk correlated with worse OS
[175]	TCGA, E-MTAB-1980	KIRC	FOXD2-AS1, NUP153-AS1, LINC02154	SUCLG2-AS1, LINC00271	higher risk score correlated with less iimmune cells infiltration and higher TME score	higher risk correlated with worse OS and DFS
[176]	TCGA	KIRC	LINC01605, AGAP2-AS1, FOXD2-AS1, LINC02195	none	higher risk score correlated with more immune check point genes expression	higher risk correlated with worse OS and clinicopathological features
[177]	TCGA	KIRC	HHLA3, H110-AS1, PICSAR, SNHG15, LINC00471, LINC02154, MINCR	LINC02027, SNHG8, EIF1B-AS1	higher risk score correlated with higher TME score and immune check point genes expression	higher risk correlated with worse OS
[152]	TCGA	HNSCC	AL359397.1, AC098679.1, AC008014.1, C6orf99, LINC01106	LINC00278, AC106820.3, AC007406.3, AC106820.5, AC022182.2, AP006621.4, TTTY14, AP006545.1, AC067930.3, DCST1 − AS1, AC022167.3, AC022098.1, AC020907.1	higher risk correlaed with CD4 memory resting T cells infiltration	higher risk correlated with shorter survival
[178]	TCGA	HNSCC	AL132800.1, AC 079,160.1, AL157888.1, SNHG16	AC021148.2, AC090587.1, AC011462.4, GRHL3-AS1	higher risk score correlated with low immune escape	higher risk correlated with worse OS and more advanced clinical stage
[179]	TCGA	HNSCC	AC004943.2, TTN-AS1, AL132800.1, WDFY3-AS2	AC090587.1, AL136419.3, AC012313.5, AC106820.5, AL162458.1, CDKN2A-DT	higher risk score correlated with lower TME score and more immune dysfunction	higher risk correlated with worse OS and clinicopathological features
[180]	TCGA	STAD	LINC01094, AC022182.1, AC011747.1, LINC02476, AC090809.1, AC084781.2, SENCR	AC005014.2, AC010422.4	higher risk score correlated with higher expression of immune check point genes	higher risk correlated with worse prognosis
[181]	TCGA	STAD	LINC01150, SNAP25-AS1, HAGLR	LINC00571	higher risk score correlated with higher immune function and expression of immune check point geens	higher risk correlated with worse OS and clinicopathological features
[182]	TCGA	BLCA	AC080023.1	AC010168.2, AC018653.3	higher risk score correlated with higher immune functions and immune cells infiltration	higher risk correlated with worse OS and clinicopathological features

CKGs have related cellular functions that interact with each other, complicating the assessment of tumor prognosis by any of these genes. CKGs have different roles among different tumors. DLD was considered as a risk factor in studies of different tumors such as uveal melanoma(UVM), glioma, and LUAD [129, 139, 143, 183]. In contrast, LIAS was analyzed as a protective factor in studies of UVM, glioma, and pancreatic adenocarcinoma(PAAD) [139, 142, 156–158]. In studies, DLAT was analyzed as a PAAD risk factor [156–158] and conversely was considered to be associated with a protective effect in glioma [142, 143]. Even in different studies on the same tumor, different investigators considered FDX1 and PDHA1 as risk or protective factor respectively [129, 130, 148, 149]. A potential association among CDKN2A, MTF1 and GLS and patient prognosis has been noted in different studies, which were believed had a consistent effect on the prognosis of patients [130, 131, 139, 156]. In one of the researches on glioma, SLC31A1 was considered a risk factor, while ATP7B was considered a protective factor [142], which further suggested that there may be a link between the copper homeostasis and cancer. Many investigators have focused on the expression levels of CKGs in different tumors and analyzed the relationship between CKGs and tumor prognosis either alone or by constructing signatures, however, the lack of biological knowledge about cuproptosis prevents direct analysis of the role of these genes in the link between cuproptosis and tumorigenesis. In addition to the expression levels and clinical significance of CKG among different tumors, the investigators also focused on the profiles of these CKGs in SNV, CNV, methylation, pathway cross-talk and so on through the pan-cancer analysis, and these genes were found to exhibit different properties in different tumors [184].

**Fig. 5:**
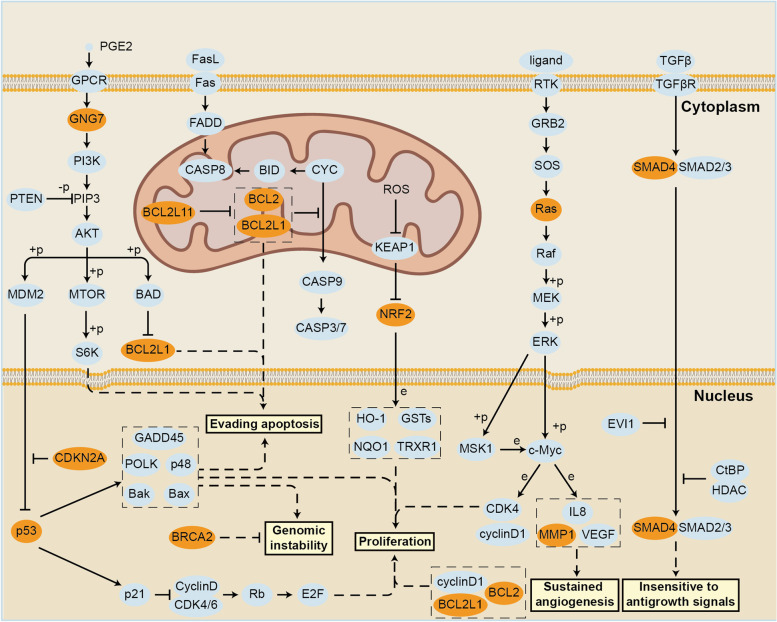
Cuproptosis related genes and cancer signaling pathways. Cuproptosis related genes occupy an important position in the tumor signaling pathways which have significant relevance to various processes of cancers, including proliferation, genomic instability, evading apoptosis, sustained angiogenesis and insensitive to antigrowth signals. The molecules linked together to accomplish the function by creating the complex, while the molecules framed by the dotted line cooperate to achieve the function. Essential genes in CRG-related cancer pathways and associated cancer processes are listed, CRGs are marked in orange

CRGs are located in several important tumor-related signaling pathways and play a critical role (Fig. 5), the exploration of which could provide possible guidance for future studies. Although so many CRGs were involved in the construction of the risk model, only a few genes were involved in the construction of different signatures, which may play a more important role. Dihydrolipoamide Branched Chain Transacylase E2(DBT) catalyzed α-keto acid to acyl-CoA, which is thought to be associated with protective effects in both LUAD and cervical cancer(CC) patients [159, 164]. Cytochrome c oxidase assembly factor 19(COX19) was involved in SCO1-dependent signaling essential for copper homeostasis [185], which was thought to be a risk factor in both gliomas and low-grade gliomas [140, 142]. Pituitary tumor-transforming gene 1 protein(PTTG1), a key regulator of the p53/TP53 pathway and DNA repair, has been considered as a risk factor in studies of HCC by different investigators [133, 135]. Surfeit 4(SURF4) is a regulator of lipoprotein export, and its high expression leads to poor prognosis of patients with BRCA [145, 147]. In addition, Enamelin(ENAM) and WD repeat domain 72(WDR72) were considered to be both associated with enamel construction in previous studies [186] and as risk or protective factors in studies on KIRC, respectively [149, 150]. MKLN1-AS and AL031985.3 were both considered risk factors for patients with hepatocellular carcinoma [136, 163], FOXD2-AS1 and LINC02154 were both considered risk factors for KIRC patients [175–177], AL513550.1 was suggested as a risk factor for CRC by different investigators [121, 172], and AL132800.1 was thought to be a risk factor for HNSCC [178, 179]. C6orf99 was regarded as a risk factor for patients with OSCC and HNSCC, two tumors with similar pathological types [152, 173]. In contrast, upregulation of AC090587.1 and AC012313 was identified to be associated with a better prognosis in HNSCC patients [178, 179], while AC073896.3 was used as a protective factor in the prognostic analysis of CRC patients [121, 172]. Certain lncRNAs were also hinted that may serve as protective or risk factors in different prognostic signatures of the same or different tumors, respectively. AC026979.4 was considered as a risk or protective factor among the different prognostic signatures of CRC patients [121, 172], and upregulation of LINC01150 was associated with better or worse prognosis in the prognostic analyses of STAD patients and CM patients [168, 181]. These possible relationships need to be verified by deeper mechanistic studies and multi-omics level analysis.

Except for the models constructed by survival analysis described above, there are also cuproptosis related signatures constructed by other methods, which was demonstrated that can also predict clinical prognosis for patients with bladder cancer and KIRC [187, 188]. As a programmed cell death, several studies have demonstrated that cuproptosis may interact with necroptosis or ferroptosis, and constructed risk models by co-opting cuproptosis-related genes with necroptosis- or ferroptosis-related genes, respectively. In a study on the prognostic risk of LGG patients, cuproptosis-related genes were analyzed by cox regression together with necroptosis-related genes, and the five genes that contributed most to the model construction were selected to construct the prognostic signature, however, these 5 genes were all considered to be necroptosis related in previous studies [189]. In the study on CRC, investigators selected cuproptosis-related genes together with ferroptosis-related genes to construct a prognostic signature, however, the contribution of each gene to the model was not described [190].

In addition to patient prognosis, the researchers have also focused on the correlation between CRGs and other cancer features like the tumor microenvironment. Less immune activation, lower levels of immune cells infiltration or worse TME scores were prevalent in patients of high-risk groups except for patients suffering gliomas [140, 141, 143, 169]. Moreover, some researchers have also focused on the expression levels of immune checkpoint genes(ICGs) such as PD-1, which are thought to associated with tumor immunosuppression and immunotherapy [191]. In most studies, ICGs were consistently highly expressed in the high-risk score group, however, the expression levels of were lower in the high-risk group of CC, BRCA and skin cutaneous melanoma(SKCM) [164, 167, 168, 174]. Most investigators performed functional enrichment analysis of CRGs based on GO and KEGG analysis, which are often highly correlated with metabolism and immune.

Although a growing number of researchers aim to construct cuproptosis-related gene signatures to predict tumor prognosis, only a few of them have been biologically validated in cell lines or clinical samples, detected by qRT-PCR, WB or IHC. Genes cited as risk factors tend to be expressed at higher levels in tumor cell lines and in patient tumor samples. However, the expression levels of risk factors in tumor cell lines and clinical tumor tissues in CRC showed inverse levels between the two [171], which may be due to the distinct tumor microenvironment of tumor cells from cell lines and clinical samples. Future studies on the cuproptosis should take note of the impact of this difference in the tumor microenviroment.

As one of the emerging therapeutic approaches, immunotherapy is often used among patients with advanced stage cancer or metastases [192]. Previous research has established that copper can modulate the expression of PD-L1, the important immune checkpoint gene crucial for immunotherapy [193], whereas the relationship between the CRGs and the efficacy of immunotherapy has been of great interest to researchers. However, most of these researchers predict the efficacy indirectly by the expression level of immune checkpoint genes or TIDE scores, only in some studies, investigators used the immunotherapy cohorts from the public databases to directly evaluate the prognosis of patients under immunotherapy. In most studies validated using immunotherapy cohorts, the low-risk group had a better prognosis for immunotherapy [152, 156, 158, 173, 194], except for those on glioma [140, 141]. Moreover, in the study on UVM, it has been found that immunotherapy was ineffective in both populations [139]. Due to the lack of biological evidence, it remains unclear what role these cuproptosis-related genes might play in the immunotherapeutic process of patients.

Some researchers have also focused on the predictive effect of cuproptosis related genetic signatures on chemotherapy efficacy. It is noteworthy that bortezomib, one of the PIs, was found to have better efficacy in different risk groups of cancers [165, 168], which further indicated the relationship between PIs and cuproptosis. However, these studies on drug sensitivity were not verified by preclinical experimental studies.

### Cuproptosis and potential cancer treatment

The discovery of the mechanism of cuproptosis provides a direction for future drug research, and copper ionophores, or called cuproptosis related drugs, that can induce the cuproptosis may have some application prospects in the future treatment of cancers [195]. ES and DSF can induce cell death by translocating copper ions into cells and mitochondria, further leading to DLAT oligomerization, reduced Fe-S stability and interaction with Npl4.

Some antimicrobial drugs also serve as copper ionophores, which inhibit the growth of microorganisms by elevating intracellular copper ion concentration. Zinc pyrithione mediated copper influx can inhibit the growth of yeast [196], 4-Br-A23187 and Dimethyldithiocarbamate can increase cell copper level and play an antibacterial role [197, 198]. Lipographic copper containing complexes with bis (thiosemicarbazone) ligands can also increase the concentration of copper ions in cancer cells and host cells of chlamydial [199, 200]. Moreover, derivatives of quinolines are also considered to have the role of copper ionophores, and their modification can change their properties for better performance [201–203]. Derivatives obtained by modification of simple compounds like 3-Hydroxyflavone [204] as well as more complex copper ionophores such as Hydrophilic Temperature-Sensitive Liposome [205] and the copper ionophore designed based on salicylaldehyde isonicotinoyl hydrazone [206] can also elevate intracellular copper levels.

Among these drugs that elevate cellular copper ion concentration, DSF and ES have received the most attention and were subjected to clinical trials. In current most clinical trials on ES and DSF, both were not found to be clinically beneficial in unselected populations but the safety of both was evaluated comprehensively (Table 3). DSF, an FDA-approved drug for the treatment of alcohol dependence, is recommended at an average dose of 125 to 500 mg per day and is well tolerated by patients [17]. ES was first developed as an anti-tumor drug, although it has not shown good effect in a past clinical trial [207], its safety has also been proven. Nanomedicines combining copper ions with copper ionophores are currently being extensively studied, and the targeting of tumors makes it possible to achieve more precise tumor killing through cuproptosis [208, 209].

**Table 3:** Clinical trials for copper ionophores

NCT Number	Status	Phases	Enrollment	Conditions	Drugs	Result	Ref
NCT00742911	Completed	Phase 1	21	solid tumors, hepatic metastases	DSF, Copper Gluconate	Disulfiram 250 mg daily was well tolerated and no objective remission was observed	[210]
NCT01907165	Completed	Early Phase 1	21	Glioblastoma	Temozolomide, DSF, Copper gluconate	Disulfiram combined with TMZ therapy has an acceptable safety profile and can improve PFS	[211]
NCT00256230	Completed	Phase 1, Phase 2	7	Stage IV Melanoma	DSF	/	Unpublished
NCT02770378	Completed	Phase 1, Phase 2	10	Glioblastoma	Temozolomide, Aprepitant, Minocycline, DSF, Celecoxib, Sertraline, Captopril, Itraconazole, Ritonavir, Auranofin	Nine drug combinations, including DSF, can be applied safely with careful monitoring	[212]
NCT03714555	Completed	Phase 2	1	Metastatic Pancreatic Cancer	nab-paclitaxel /gemcitabine Protocol Plus DSF/Copper Gluconate FOLFIRINOX regimen Plus DSF/Copper Gluconate Single-agent gemcitabine regimen Plus DSF/Copper Gluconate	/	Unpublished
NCT02101008	Completed	Phase 2	12	Melanoma	DSF and chelated zinc	/	Unpublished
NCT03034135	Completed	Phase 2	23	Recurrent Glioblastoma	DSF/Copper, Temozolomide (TMZ)	DSF combined with TMZ treatment is well tolerated but has limited effect on unselected populations	[213]
NCT02678975	Completed	Phase 2, Phase 3	88	Glioma, Glioblastoma	DSF, Copper, Alkylating Agents	DSF combined with Alkylating treatment has limited risk profile	[214]
NCT00312819	Completed	Phase 2, Phase 3	60	Non-small Cell Lung Cancer	chemotherapy ± DSF	DSF in combination with cisplatin and vincristine is well tolerated and may prolong patient survival	[215]
NCT01118741	Completed	/	19	Prostate Cancer	DSF	DSF treatment is tolerated but has no clinical benefit	[216]
NCT00571116	Terminated	Phase 1	9	Metastatic Melanoma	DSF, Arsenic trioxide	/	Unpublished
NCT02963051	Terminated	Phase 1	9	Prostate Cancer	Copper, DSF, Copper gluconate	No significant PSA decline with imaging response was observed	[217]
NCT03151772	Terminated	Early Phase 1	3	Glioblastoma	DSF, Metformin	/	Unpublished
NCT00808418	Completed	Phase 1	34	Prostate Cancer	ES, Docetaxel	/	Unpublished
NCT00088114	Completed	Phase 1	50	Neoplasms	ES, paclitaxel	/	Unpublished
NCT00827203	Suspended	Phase 1	30	Metastatic Solid Tumors	ES	ES treatment in combination with paclitaxel was well tolerated, with a toxicity profile consistent with that of paclitaxel alone	[218]
NCT00084214	Completed	Phase 1, Phase 2	103	Melanoma	ES, Paclitaxel	ES combined with paclitaxel treatment doubled median PFS with an acceptable toxicity profile	[219]
NCT00088088	Completed	Phase 1, Phase 2	86	Stage IIIB/IV Non-Small Cell Lung Cancer	Paclitaxel, Carboplatin, ES	/	Unpublished
NCT00888615	Completed	Phase 2	58	Primary and recurrent fallopian tube cancer, primary and recurrent ovarian cancer, primary and recurrent primary peritoneal cancer	ES, Paclitaxel	ES combined with paclitaxel was well tolerated, but its response rate was inadequate	[220]
NCT00087997	Completed	Phase 2	80	Soft Tissue Sarcoma	ES	ES enhances the efficacy of taxane through the action of HSP70	[221]
NCT00522834	Terminated	Phase 3	630	Melanoma	ES, Paclitaxel	ES combined with paclitaxel did not significantly improve PFS, and combination therapy improved PFS in patients with normal serum LDH levels	[207]

There is a significant correlation between cuproptosis caused by copper ionophores and the level of mitochondrial metabolism, which should be considered comprehensively during future studies of exploring possible drugs that rely on cuproptosis for cancers treatment. Certain tumors inherently exhibit higher levels of mitochondrial metabolism, such as melanoma, breast cancer and leukemia [5]. Some cancer stem cell-like cells among cancers like glioblastoma [7] and cholangiocarcinoma [8] also exhibit higher levels of aerobic respiration. some drug-resistant tumors exhibit a high mitochondrial metabolic state [9–13]. Higher levels of mitochondrial respiration were demonstrated in certain drug-resistant tumors treated with chemotherapy by cisplatin [9] or 5-fluorouracil [11] or target therapy by anti-EGFR [12] or anti-BCL-2 [13], which were also thought to be associated with drug resistance. Therefore, copper ionophores may be used in combination with small molecule targeting agents that act on EGFR or BCL-2 to achieve better clinical outcomes, which needs further clinical trials. Tumor cells treated with certain antitumor drugs such as PI have also been found to promote the transformation of tumor cells to a high mitochondrial metabolic state [14, 15], combination with which may lead to better results in cuproptosis related therapy. In conclusion, copper ionophores may be more effective in tumors with higher levels of mitochondrial metabolism, and these patients may be the potential beneficiaries of future cuproptosis-inducing therapy, and treatment by other drugs inducing high mitochondrial respiratory state of the tumor in combination with copper ionophores is also a potential therapeutic direction. In the phase III clinical trial of ES, there was no statistical difference in efficacy between the experimental and control groups. However, among patients with low serum LDH levels the effect of ES differed between the two groups [207]. In future practical clinical use, serum LDH levels may be used as an indicator of whether or not to treat with cuproptosis related drugs and to determine the likely efficacy of these drugs. Moreover, Researchers are also focused on investigating new copper ionophores as drugs to target tumors [222]. Copper ionophores have different physicochemical properties and may be used in cancers with different characteristics. In addition to copper ionophores, copper complexes can also increase intracellular copper ion concentrations leading to cancer cell death [84], may also be used in the future as a cuproptosis-related treatment. Also noteworthy in the development of copper ionophores for clinical therapeutic use is the fact that relatively small changes in their structure can cause changes in properties and functions, like the different derivatives of bis(N4-methylthiosemicarbazone) [223, 224] or quinolines [225, 226]. In conclusion, copper ionophores can be used in combination with targeted therapeutic agents such as TKI and PI, which should be used in tumors with a high mitochondrial metabolic status, and LDH may be used as a predictor and prognostic indicator to guide treatment before and after drug administration, respectively. Further work is required to determine the viability of using cuproptosis-related therapy in certain patients with specific cancers.

## Conclusion and future perspectives

Copper is essential for cell life, yet its excess has also been found to cause cell death [37]. As the concept of cuproptosis was introduced, a large number of publications in the form of research highlight described it [1, 2]. Some researcher also focused on copper ionophores which played an important role in the discovery of cuproptosis and reviewed them [71]. However, there is still no review systematically addressing this field.

The discovery of the copper death concept relied on the study of copper ionophores that have antitumor effects. Among the studies on copper ionophores, it is commonly observed that these ionophores cause cell death by importing copper [74]. Yet, the mechanism of copper-induced cell death [7, 14, 82] and the exact manner in which it occurs [72, 73, 89, 90] has been controversial in the past until the convincing mechanism of cuproptosis was proposed in 2022 [4]. Cuproptosis was described that showed close relationship with mitochondrial respiration level and LA pathway. However, the mechanism of cuproptosis remains to be further explored. On the one hand, the specific pathways of action of key factors such as FDX1 remain unexplored. On the other hand, certain mechanism through which cuproptosis is inhibited among normal cells have not been clearly described. Furthermore, the relationship between several possible mechanisms thought to contribute to copper induced cell death in past studies also need to be further clarified [14, 81, 82]. Finally, characteristic changes in cells undergoing cuproptosis at the cellular morphological and molecular levels have also not been described.

With the introduction of the concept of cuproptosis, an increasing number of researchers are intent on exploring its relationship with tumors. Studies on the relationship have covered most common cancer types and analyzed the links between CRGs and various aspects of tumor characteristics. However, these studies have only indirectly demonstrated the links between cuproptosis and cancer due to the insufficient biological evidence and experimental validation, whether these genes play a direct role in the relationship between cuproptosis and tumors or receive indirect effects from both is still unknown. Several genes have been identified by different investigators in these studies to construct signatures that may play a more significant role in the association between cuproptosis and cancers. Further studies, which examine the relationship between these repeatedly mentioned CRGs and cuproptosis, will need to be undertaken.

Past studies on copper have also provided a sufficient basis for future studies on the specific mechanisms of cuproptosis and its relationship with tumors. A variety of drugs have been identified in past studies to elevate intracellular copper ion concentrations including the aforementioned copper ionophores, copper ion complexes, and many copper chelators that block the elevation of copper ion concentrations such as triethylenetetramine dihydrochloride [227], 8-hydroxyquinoline [228], penicillamine [229], methanobactin [230], Bathocuproine disulphonate [231] and choline tetrathiomolybdate [232]. Through these substances, the intracellular concentration of copper ions can be regulated to induce or suppress the occurrence of cuproptosis. Researchers have been always focusing on their clinical antitumor effects [207], and their safety has been verified by sufficient clinical trials. In future cuproptosis-inducing therapies, patient populations that may be benefited should be preemptively evaluated, as certain cancers with higher levels of mitochondrial metabolism [5], certain cancers with tumor stem cell-like cells [7, 8], and certain drug-resistant cancers may have better efficacy. Proteasome inhibitors [14] can induce a high mitochondrial metabolic state in cells, with which the combination of copper ionophores for tumor therapy is also a possible future application direction. On the one hand, the concept of cuproptosis has brought the current investigations on the mechanism of copper-induced cell death to a new milestone, and there is sufficient basement for the future mechanistic studies on cuproptosis. On the other hand, there has been a large amount of exploration on the indirect relationship between cuproptosis and tumors, which provides guidance for future studies on the direct relationship of both. In summary, it has been shown that cuproptosis is an independent and novel cell death pattern. However, the specific mechanism by which cuproptosis occurred and the links between cuproptosis and cancer still needs further study.

## Data Availability

Not applicable.

## Abbreviations

Acetyl-CoA: Acetyl coenzyme A
ARID1A: AT-rich interaction domain 1A
ATG13: Autophagy related 13
ATK: Agammaglobulinaemia tyrosine kinase
ATOX1: Antioxidant 1 copper chaperone
ATP7A: ATPase copper taransporting alpha
ATP7B: ATPase copper transporting beta
BRAF: B-Raf proto-oncogene
BRCA: Breast cancer
CC: Cervical cancer
CCS: Copper chaperone for superoxide dismutase
CDKN2A: Cyclin dependent kinase inhibitor 2A
CESC: Cervical squamous cell carcinoma
CKGs: Cuproptosis key genes
COA6: Cytochrome c oxidase assembly factor 6
COX: Cytochrome c oxidase
COX19: Cytochrome c oxidase assembly factor 19
CP: Ceruloplasmin
CRC: Colorectal cancer
CRGs: Cuproptosis related genes
CTR1: Copper transporter 1
DBT: Dihydrolipoamide branched chain transacylase E2
DFS: Disease free survival
DLAT: Dihydrolipoamide s-acetyltransferase
DLD: Dihydrolipoamide dehydrogenase
DSF: Disulfiram
ENAM: Enamelin
ERK: Extracellular regulated protein kinases
ES: Elesclomol
ETC: Electron transport chain
FDX1: Ferredoxin 1
Fe-S cluster: Iron-sulfur cluster
FoxO1a: Forkhead box O1a
FoxO4: Forkhead box O4
GBM: Glioblastoma
GLS: Glutaminase
GSH: Glutathione
HCC: Hepatocellular carcinoma
HIF-1: Hypoxia inducible factor 1
HIF-1α: Hypoxia inducible factor 1 subunit alpha
HNSC: Head and neck squamous cell carcinoma
ICGs: Immune checkpoint genes
JNK: C-Jun N-terminal kinase
KIRC: Clear cell renal cell carcinoma
LA: Lipoic acid
LGG: Low grade glioma
LIAS: Lipoic acid synthetase
LIPT1: Lipoyltransferase 1
LUAD: Lung adenocarcinoma
MAPK: Mitogen-activated protein kinase
MEK1: Mitogen-activated proteinkinase kinase 1
MT1/2: Metallothionein1/2
MTF1: Metal regulatory transcription factor 1
NAC: N-Acetylcysteine
OS: Overall survival
PAAD: Pancreatic adenocarcinoma
PDC: Pyruvate dehydrogenase complex
PDE3B: Phosphodiesterase 3B
PDHA1: Pyruvate dehydrogenase e1 subunit alpha 1
PDHB: Pyruvate dehydrogenase e1 subunit beta
PI: Proteasome inhibitors
PI3K: Phosphoinositide-3-kinase
PTTG1: Pituitary tumor-transforming gene 1 protein
ROS: Reactive oxygen species
RTK: Receptor tyrosine kinase
SCO1/2: Cytochrome c oxidase1/2
SKCM: Skin cutaneous melanoma
SLC25A3: Solute carrier family 25 member 3
SLC31A1: Solute carrier family 31 member 1
SOD1: Superoxide dismutase 1
SURF4: Surfeit 4
TTM: Tetrathiomolybdate
ULK: Unc-51 like autophagy activating kinase
UVM: Uveal melanoma
VEGF: Vascular endothelial growth factor
WDR72: WD repeat domain 72
XIAP: X-linked inhibitor of apoptosis
